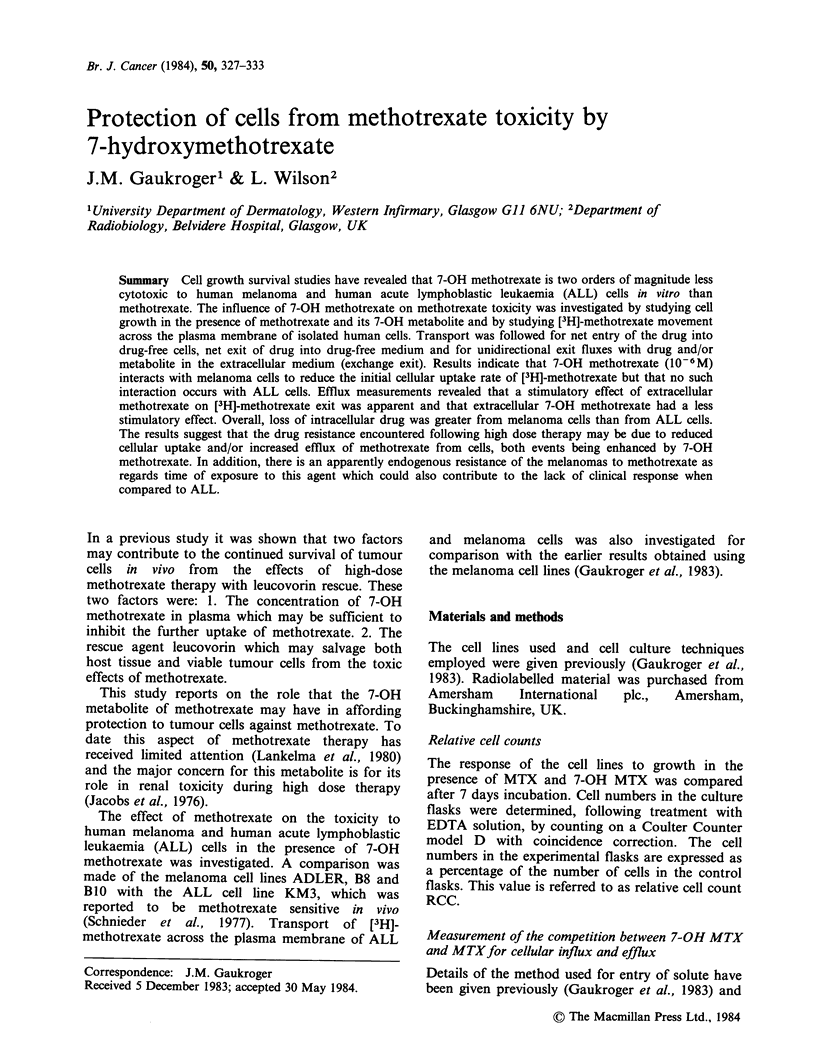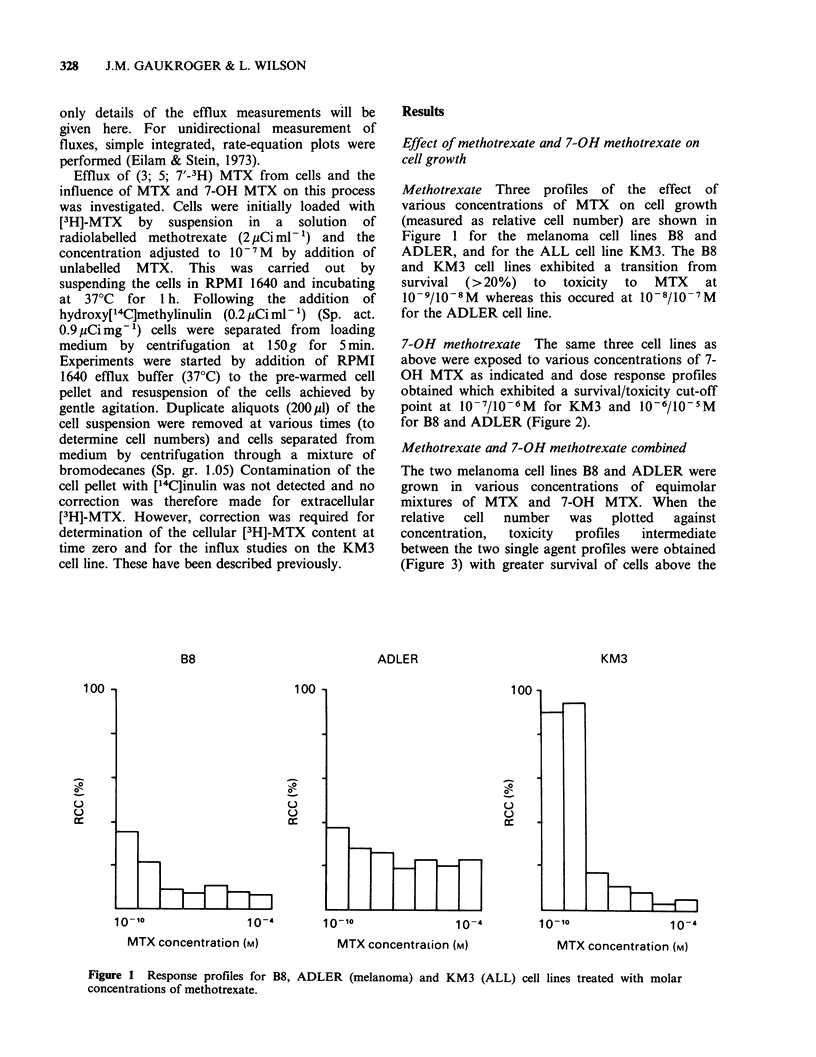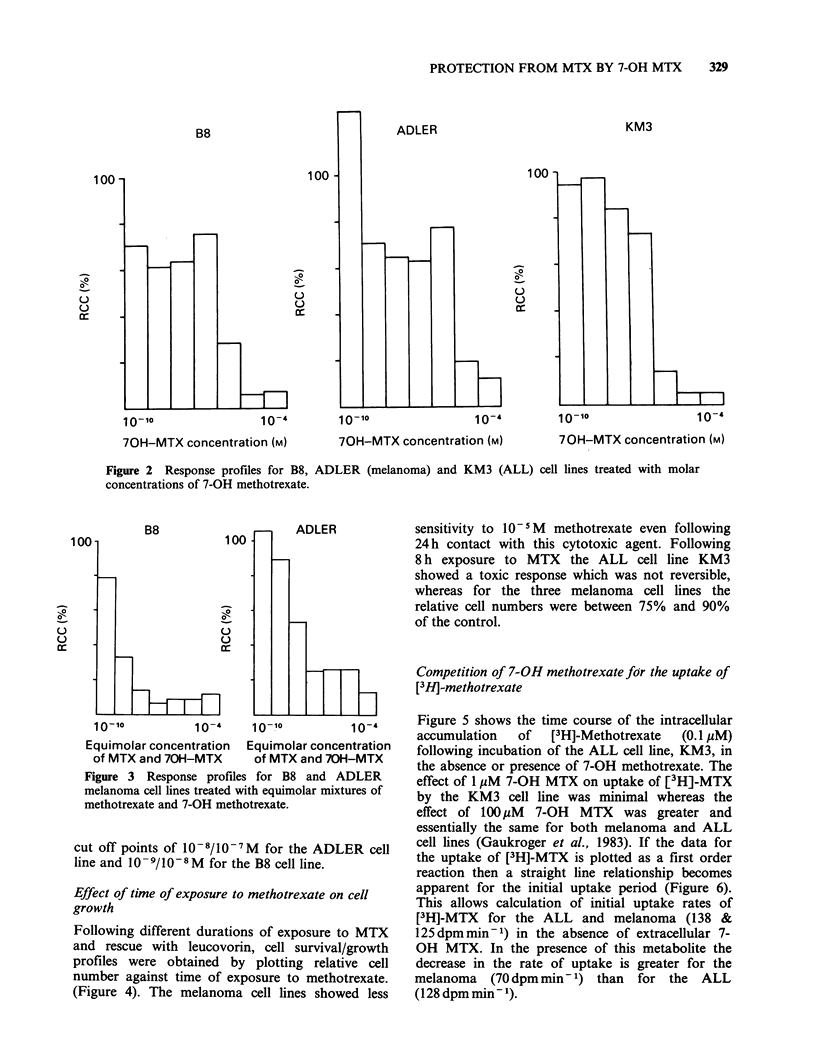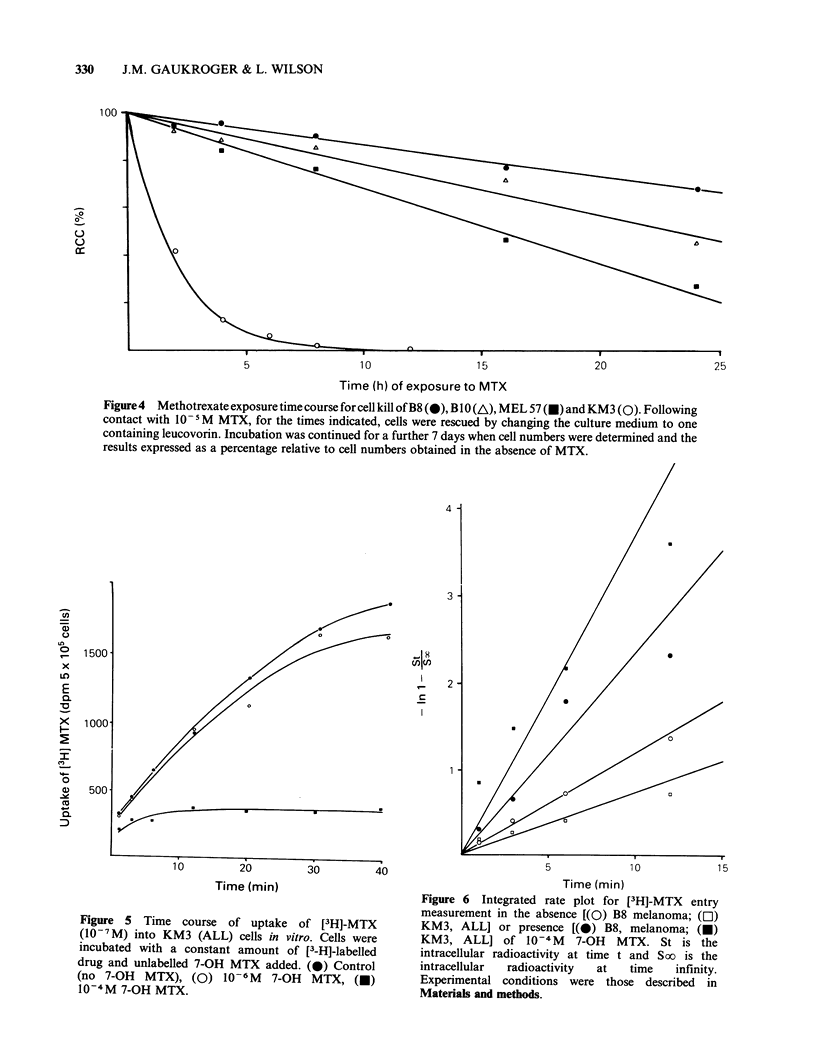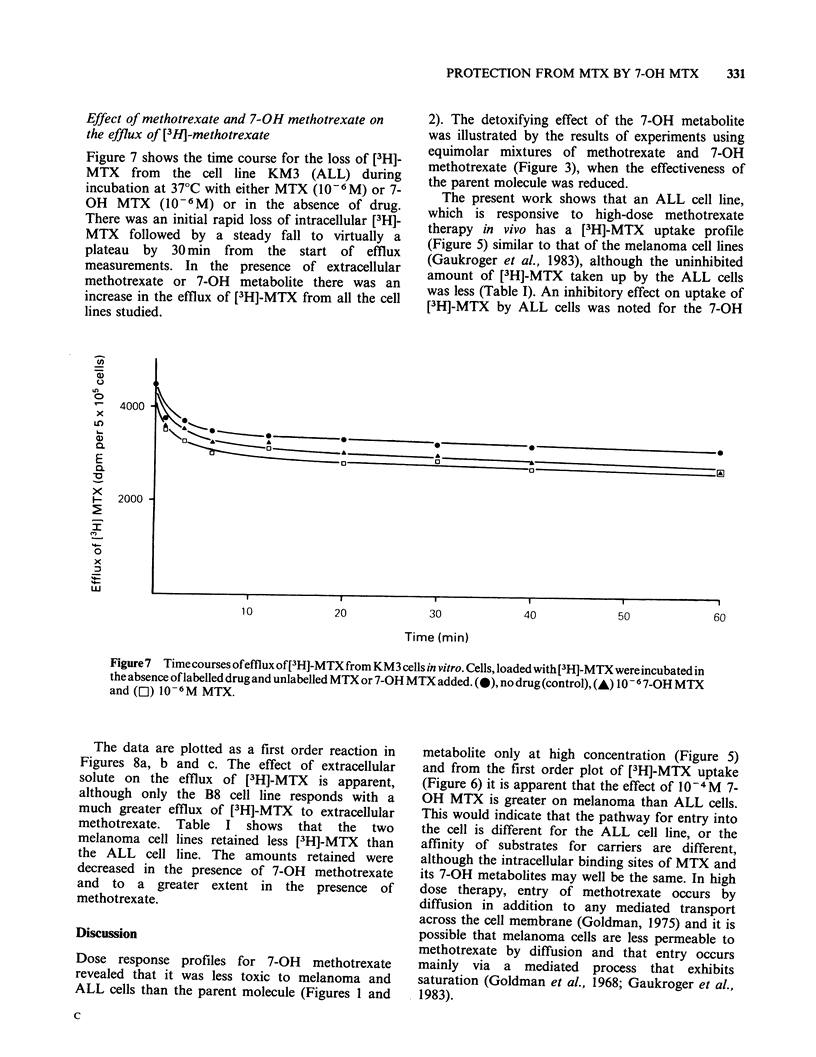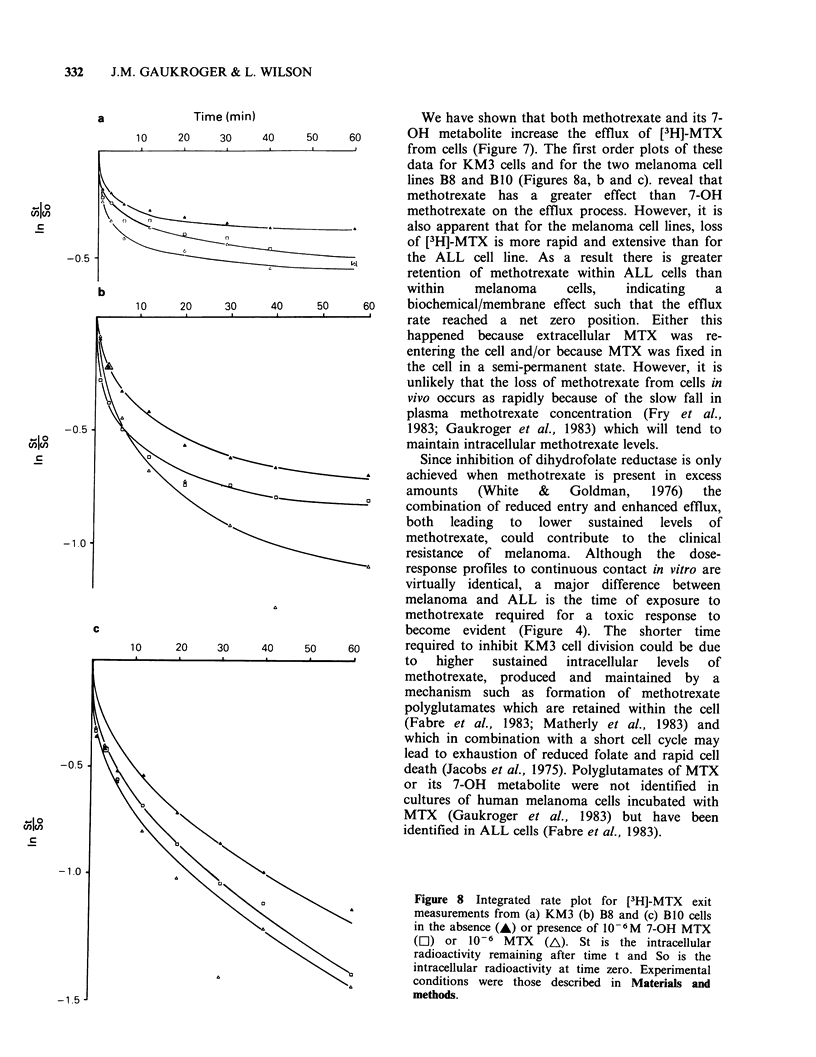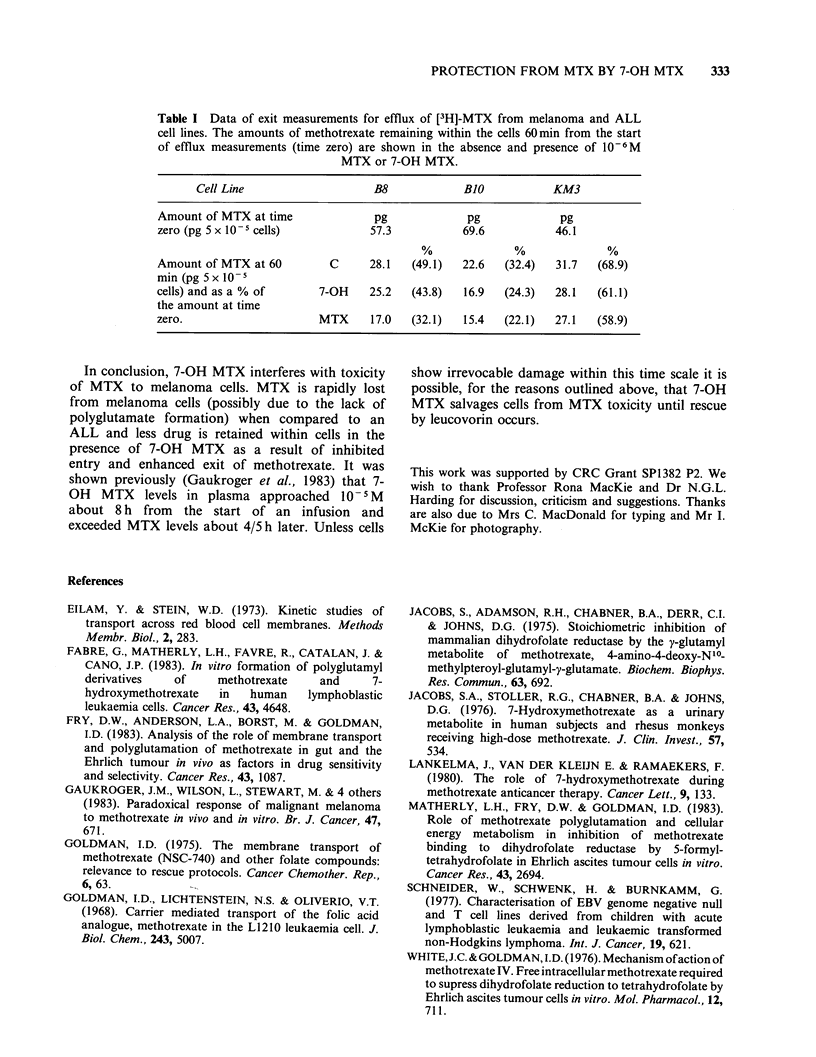# Protection of cells from methotrexate toxicity by 7-hydroxymethotrexate.

**DOI:** 10.1038/bjc.1984.179

**Published:** 1984-09

**Authors:** J. M. Gaukroger, L. Wilson

## Abstract

Cell growth survival studies have revealed that 7-OH methotrexate is two orders of magnitude less cytotoxic to human melanoma and human acute lymphoblastic leukaemia (ALL) cells in vitro than methotrexate. The influence of 7-OH methotrexate on methotrexate toxicity was investigated by studying cell growth in the presence of methotrexate and its 7-OH metabolite and by studying [3H]-methotrexate movement across the plasma membrane of isolated human cells. Transport was followed for net entry of the drug into drug-free cells, net exit of drug into drug-free medium and for unidirectional exit fluxes with drug and/or metabolite in the extracellular medium (exchange exit). Results indicate that 7-OH methotrexate (10(-6) M) interacts with melanoma cells to reduce the initial cellular uptake rate of [3H]-methotrexate but that no such interaction occurs with ALL cells. Efflux measurements revealed that a stimulatory effect of extracellular methotrexate on [3H]-methotrexate exit was apparent and that extracellular 7-OH methotrexate had a less stimulatory effect. Overall, loss of intracellular drug was greater from melanoma cells than from ALL cells. The results suggest that the drug resistance encountered following high dose therapy may be due to reduced cellular uptake and/or increased efflux of methotrexate from cells, both events being enhanced by 7-OH methotrexate. In addition, there is an apparently endogenous resistance of the melanomas to methotrexate as regards time of exposure to this agent which could also contribute to the lack of clinical response when compared to ALL.


*Br. J. Cancer* (1984), **50**, 327–333

### Protection of cells from methotrexate toxicity by 7-hydroxymethotrexate

J.M. Gaukroger[1] & L. Wilson[2]

[1]*University Department of Dermatology, Western Infirmary, Glasgow G11 6NU;* [2]*Department of Radiobiology, Belvidere Hospital, Glasgow, UK*

**Summary** Cell growth survival studies have revealed that 7-OH methotrexate is two orders of magnitude less cytotoxic to human melanoma and human acute lymphoblastic leukaemia (ALL) cells *in vitro* than methotrexate. The influence of 7-OH methotrexate on methotrexate toxicity was investigated by studying cell growth in the presence of methotrexate and its 7-OH metabolite and by studying [$^3$H]-methotrexate movement across the plasma membrane of isolated human cells. Transport was followed for net entry of the drug into drug-free cells, net exit of drug into drug-free medium and for unidirectional exit fluxes with drug and/or metabolite in the extracellular medium (exchange exit). Results indicate that 7-OH methotrexate ($10^{-6}$M) interacts with melanoma cells to reduce the initial cellular uptake rate of [$^3$H]-methotrexate but that no such interaction occurs with ALL cells. Efflux measurements revealed that a stimulatory effect of extracellular methotrexate on [$^3$H]-methotrexate exit was apparent and that extracellular 7-OH methotrexate had a less stimulatory effect. Overall, loss of intracellular drug was greater from melanoma cells than from ALL cells. The results suggest that the drug resistance encountered following high dose therapy may be due to reduced cellular uptake and/or increased efflux of methotrexate from cells, both events being enhanced by 7-OH methotrexate. In addition, there is an apparently endogenous resistance of the melanomas to methotrexate as regards time of exposure to this agent which could also contribute to the lack of clinical response when compared to ALL.

In a previous study it was shown that two factors may contribute to the continued survival of tumour cells *in vivo* from the effects of high-dose methotrexate therapy with leucovorin rescue. These two factors were: 1. The concentration of 7-OH methotrexate in plasma which may be sufficient to inhibit the further uptake of methotrexate. 2. The rescue agent leucovorin which may salvage both host tissue and viable tumour cells from the toxic effects of methotrexate.

This study reports on the role that the 7-OH metabolite of methotrexate may have in affording protection to tumour cells against methotrexate. To date this aspect of methotrexate therapy has received limited attention (Lankelma *et al.*, 1980) and the major concern for this metabolite is for its role in renal toxicity during high dose therapy (Jacobs *et al.*, 1976).

The effect of methotrexate on the toxicity to human melanoma and human acute lymphoblastic leukaemia (ALL) cells in the presence of 7-OH methotrexate was investigated. A comparison was made of the melanoma cell lines ADLER, B8 and B10 with the ALL cell line KM3, which was reported to be methotrexate sensitive *in vivo* (Schnieder *et al.*, 1977). Transport of [$^3$H]-methotrexate across the plasma membrane of ALL and melanoma cells was also investigated for comparison with the earlier results obtained using the melanoma cell lines (Gaukroger *et al.*, 1983).

Correspondence: J.M. Gaukroger
Received 5 December 1983; accepted 30 May 1984.

### Materials and methods

The cell lines used and cell culture techniques employed were given previously (Gaukroger *et al.*, 1983). Radiolabelled material was purchased from Amersham International plc., Amersham, Buckinghamshire, UK.

### *Relative cell counts*

The response of the cell lines to growth in the presence of MTX and 7-OH MTX was compared after 7 days incubation. Cell numbers in the culture flasks were determined, following treatment with EDTA solution, by counting on a Coulter Counter model D with coincidence correction. The cell numbers in the experimental flasks are expressed as a percentage of the number of cells in the control flasks. This value is referred to as relative cell count RCC.

### *Measurement of the competition between 7-OH MTX and MTX for cellular influx and efflux*

Details of the method used for entry of solute have been given previously (Gaukroger *et al.*, 1983) and

© The Macmillan Press Ltd., 1984

only details of the efflux measurements will be given here. For unidirectional measurement of fluxes, simple integrated, rate-equation plots were performed (Eilam & Stein, 1973).

Efflux of (3; 5; 7′-$^3$H) MTX from cells and the influence of MTX and 7-OH MTX on this process was investigated. Cells were initially loaded with [$^3$H]-MTX by suspension in a solution of radiolabelled methotrexate ($2\,\mu$Ci ml$^{-1}$) and the concentration adjusted to $10^{-7}$M by addition of unlabelled MTX. This was carried out by suspending the cells in RPMI 1640 and incubating at 37°C for 1 h. Following the addition of hydroxy[$^{14}$C]methylinulin ($0.2\,\mu$Ci ml$^{-1}$) (Sp. act. $0.9\,\mu$Ci mg$^{-1}$) cells were separated from loading medium by centrifugation at 150*g* for 5 min. Experiments were started by addition of RPMI 1640 efflux buffer (37°C) to the pre-warmed cell pellet and resuspension of the cells achieved by gentle agitation. Duplicate aliquots (200 $\mu$l) of the cell suspension were removed at various times (to determine cell numbers) and cells separated from medium by centrifugation through a mixture of bromodecanes (Sp. gr. 1.05) Contamination of the cell pellet with [$^{14}$C]inulin was not detected and no correction was therefore made for extracellular [$^3$H]-MTX. However, correction was required for determination of the cellular [$^3$H]-MTX content at time zero and for the influx studies on the KM3 cell line. These have been described previously.

### Results

### *Effect of methotrexate and 7-OH methotrexate on cell growth*

*Methotrexate* Three profiles of the effect of various concentrations of MTX on cell growth (measured as relative cell number) are shown in Figure 1 for the melanoma cell lines B8 and ADLER, and for the ALL cell line KM3. The B8 and KM3 cell lines exhibited a transition from survival (>20%) to toxicity to MTX at $10^{-9}/10^{-8}$M whereas this occured at $10^{-8}/10^{-7}$M for the ADLER cell line.

*7-OH methotrexate* The same three cell lines as above were exposed to various concentrations of 7-OH MTX as indicated and dose response profiles obtained which exhibited a survival/toxicity cut-off point at $10^{-7}/10^{-6}$M for KM3 and $10^{-6}/10^{-5}$M for B8 and ADLER (Figure 2).

### *Methotrexate and 7-OH methotrexate combined*

The two melanoma cell lines B8 and ADLER were grown in various concentrations of equimolar mixtures of MTX and 7-OH MTX. When the relative cell number was plotted against concentration, toxicity profiles intermediate between the two single agent profiles were obtained (Figure 3) with greater survival of cells above the

**Figure 1** Response profiles for B8, ADLER (melanoma) and KM3 (ALL) cell lines treated with molar concentrations of methotrexate.

**Figure 2** Response profiles for B8, ADLER (melanoma) and KM3 (ALL) cell lines treated with molar concentrations of 7-OH methotrexate.

**Figure 3** Response profiles for B8 and ADLER melanoma cell lines treated with equimolar mixtures of methotrexate and 7-OH methotrexate.

cut off points of $10^{-8}/10^{-7}$ M for the ADLER cell line and $10^{-9}/10^{-8}$ M for the B8 cell line.

### *Effect of time of exposure to methotrexate on cell growth*

Following different durations of exposure to MTX and rescue with leucovorin, cell survival/growth profiles were obtained by plotting relative cell number against time of exposure to methotrexate. (Figure 4). The melanoma cell lines showed less sensitivity to $10^{-5}$ M methotrexate even following 24 h contact with this cytotoxic agent. Following 8 h exposure to MTX the ALL cell line KM3 showed a toxic response which was not reversible, whereas for the three melanoma cell lines the relative cell numbers were between 75% and 90% of the control.

### *Competition of 7-OH methotrexate for the uptake of [$^3$H]-methotrexate*

Figure 5 shows the time course of the intracellular accumulation of [$^3$H]-Methotrexate (0.1 $\mu$M) following incubation of the ALL cell line, KM3, in the absence or presence of 7-OH methotrexate. The effect of 1 $\mu$M 7-OH MTX on uptake of [$^3$H]-MTX by the KM3 cell line was minimal whereas the effect of 100 $\mu$M 7-OH MTX was greater and essentially the same for both melanoma and ALL cell lines (Gaukroger *et al.*, 1983). If the data for the uptake of [$^3$H]-MTX is plotted as a first order reaction then a straight line relationship becomes apparent for the initial uptake period (Figure 6). This allows calculation of initial uptake rates of [$^3$H]-MTX for the ALL and melanoma (138 & 125 dpm min$^{-1}$) in the absence of extracellular 7-OH MTX. In the presence of this metabolite the decrease in the rate of uptake is greater for the melanoma (70 dpm min$^{-1}$) than for the ALL (128 dpm min$^{-1}$).

**Figure 4** Methotrexate exposure time course for cell kill of B8 (●), B10 (△), MEL 57 (■) and KM3 (○). Following contact with $10^{-5}$ M MTX, for the times indicated, cells were rescued by changing the culture medium to one containing leucovorin. Incubation was continued for a further 7 days when cell numbers were determined and the results expressed as a percentage relative to cell numbers obtained in the absence of MTX.

**Figure 5** Time course of uptake of [$^3$H]-MTX ($10^{-7}$ M) into KM3 (ALL) cells *in vitro*. Cells were incubated with a constant amount of [$^3$-H]-labelled drug and unlabelled 7-OH MTX added. (●) Control (no 7-OH MTX), (○) $10^{-6}$ M 7-OH MTX, (■) $10^{-4}$ M 7-OH MTX.

**Figure 6** Integrated rate plot for [$^3$H]-MTX entry measurement in the absence [(○) B8 melanoma; (□) KM3, ALL] or presence [(●) B8, melanoma; (■) KM3, ALL] of $10^{-4}$ M 7-OH MTX. St is the intracellular radioactivity at time t and S∞ is the intracellular radioactivity at time infinity. Experimental conditions were those described in **Materials and methods**.

*Effect of methotrexate and 7-OH methotrexate on the efflux of [³H]-methotrexate*

Figure 7 shows the time course for the loss of [³H]-MTX from the cell line KM3 (ALL) during incubation at 37°C with either MTX ($10^{-6}$M) or 7-OH MTX ($10^{-6}$M) or in the absence of drug. There was an initial rapid loss of intracellular [³H]-MTX followed by a steady fall to virtually a plateau by 30 min from the start of efflux measurements. In the presence of extracellular methotrexate or 7-OH metabolite there was an increase in the efflux of [³H]-MTX from all the cell lines studied.

**Figure 7** Time courses of efflux of [³H]-MTX from KM3 cells *in vitro*. Cells, loaded with [³H]-MTX were incubated in the absence of labelled drug and unlabelled MTX or 7-OH MTX added. (●), no drug (control), (▲) $10^{-6}$ 7-OH MTX and (□) $10^{-6}$M MTX.

The data are plotted as a first order reaction in Figures 8a, b and c. The effect of extracellular solute on the efflux of [³H]-MTX is apparent, although only the B8 cell line responds with a much greater efflux of [³H]-MTX to extracellular methotrexate. Table I shows that the two melanoma cell lines retained less [³H]-MTX than the ALL cell line. The amounts retained were decreased in the presence of 7-OH methotrexate and to a greater extent in the presence of methotrexate.

### Discussion

Dose response profiles for 7-OH methotrexate revealed that it was less toxic to melanoma and ALL cells than the parent molecule (Figures 1 and 2). The detoxifying effect of the 7-OH metabolite was illustrated by the results of experiments using equimolar mixtures of methotrexate and 7-OH methotrexate (Figure 3), when the effectiveness of the parent molecule was reduced.

The present work shows that an ALL cell line, which is responsive to high-dose methotrexate therapy *in vivo* has a [³H]-MTX uptake profile (Figure 5) similar to that of the melanoma cell lines (Gaukroger *et al.*, 1983), although the uninhibited amount of [³H]-MTX taken up by the ALL cells was less (Table I). An inhibitory effect on uptake of [³H]-MTX by ALL cells was noted for the 7-OH metabolite only at high concentration (Figure 5) and from the first order plot of [³H]-MTX uptake (Figure 6) it is apparent that the effect of $10^{-4}$M 7-OH MTX is greater on melanoma than ALL cells. This would indicate that the pathway for entry into the cell is different for the ALL cell line, or the affinity of substrates for carriers are different, although the intracellular binding sites of MTX and its 7-OH metabolites may well be the same. In high dose therapy, entry of methotrexate occurs by diffusion in addition to any mediated transport across the cell membrane (Goldman, 1975) and it is possible that melanoma cells are less permeable to methotrexate by diffusion and that entry occurs mainly via a mediated process that exhibits saturation (Goldman *et al.*, 1968; Gaukroger *et al.*, 1983).

We have shown that both methotrexate and its 7-OH metabolite increase the efflux of [$^3$H]-MTX from cells (Figure 7). The first order plots of these data for KM3 cells and for the two melanoma cell lines B8 and B10 (Figures 8a, b and c). reveal that methotrexate has a greater effect than 7-OH methotrexate on the efflux process. However, it is also apparent that for the melanoma cell lines, loss of [$^3$H]-MTX is more rapid and extensive than for the ALL cell line. As a result there is greater retention of methotrexate within ALL cells than within melanoma cells, indicating a biochemical/membrane effect such that the efflux rate reached a net zero position. Either this happened because extracellular MTX was re-entering the cell and/or because MTX was fixed in the cell in a semi-permanent state. However, it is unlikely that the loss of methotrexate from cells *in vivo* occurs as rapidly because of the slow fall in plasma methotrexate concentration (Fry *et al.*, 1983; Gaukroger *et al.*, 1983) which will tend to maintain intracellular methotrexate levels.

Since inhibition of dihydrofolate reductase is only achieved when methotrexate is present in excess amounts (White & Goldman, 1976) the combination of reduced entry and enhanced efflux, both leading to lower sustained levels of methotrexate, could contribute to the clinical resistance of melanoma. Although the dose-response profiles to continuous contact *in vitro* are virtually identical, a major difference between melanoma and ALL is the time of exposure to methotrexate required for a toxic response to become evident (Figure 4). The shorter time required to inhibit KM3 cell division could be due to higher sustained intracellular levels of methotrexate, produced and maintained by a mechanism such as formation of methotrexate polyglutamates which are retained within the cell (Fabre *et al.*, 1983; Matherly *et al.*, 1983) and which in combination with a short cell cycle may lead to exhaustion of reduced folate and rapid cell death (Jacobs *et al.*, 1975). Polyglutamates of MTX or its 7-OH metabolite were not identified in cultures of human melanoma cells incubated with MTX (Gaukroger *et al.*, 1983) but have been identified in ALL cells (Fabre *et al.*, 1983).

**Figure 8** Integrated rate plot for [$^3$H]-MTX exit measurements from (a) KM3 (b) B8 and (c) B10 cells in the absence (▲) or presence of $10^{-6}$ M 7-OH MTX (□) or $10^{-6}$ MTX (△). St is the intracellular radioactivity remaining after time t and So is the intracellular radioactivity at time zero. Experimental conditions were those described in **Materials and methods**.

**Table I** Data of exit measurements for efflux of [$^3$H]-MTX from melanoma and ALL cell lines. The amounts of methotrexate remaining within the cells 60 min from the start of efflux measurements (time zero) are shown in the absence and presence of $10^{-6}$ M MTX or 7-OH MTX.

| *Cell Line* | | *B8* | | *B10* | | *KM3* | |
|---|---|---|---|---|---|---|---|
| Amount of MTX at time zero (pg $5 \times 10^{-5}$ cells) | | pg 57.3 | | pg 69.6 | | pg 46.1 | |
| | | | % | | % | | % |
| Amount of MTX at 60 min (pg $5 \times 10^{-5}$ cells) and as a % of the amount at time zero. | C | 28.1 | (49.1) | 22.6 | (32.4) | 31.7 | (68.9) |
| | 7-OH | 25.2 | (43.8) | 16.9 | (24.3) | 28.1 | (61.1) |
| | MTX | 17.0 | (32.1) | 15.4 | (22.1) | 27.1 | (58.9) |

In conclusion, 7-OH MTX interferes with toxicity of MTX to melanoma cells. MTX is rapidly lost from melanoma cells (possibly due to the lack of polyglutamate formation) when compared to an ALL and less drug is retained within cells in the presence of 7-OH MTX as a result of inhibited entry and enhanced exit of methotrexate. It was shown previously (Gaukroger *et al.*, 1983) that 7-OH MTX levels in plasma approached $10^{-5}$ M about 8 h from the start of an infusion and exceeded MTX levels about 4/5 h later. Unless cells show irrevocable damage within this time scale it is possible, for the reasons outlined above, that 7-OH MTX salvages cells from MTX toxicity until rescue by leucovorin occurs.

This work was supported by CRC Grant SP1382 P2. We wish to thank Professor Rona MacKie and Dr N.G.L. Harding for discussion, criticism and suggestions. Thanks are also due to Mrs C. MacDonald for typing and Mr I. McKie for photography.

### References

EILAM, Y. & STEIN, W.D. (1973). Kinetic studies of transport across red blood cell membranes. *Methods Membr. Biol.*, **2**, 283.

FABRE, G., MATHERLY, L.H., FAVRE, R., CATALAN, J. & CANO, J.P. (1983). *In vitro* formation of polyglutamyl derivatives of methotrexate and 7-hydroxymethotrexate in human lymphoblastic leukaemia cells. *Cancer Res.*, **43**, 4648.

FRY, D.W., ANDERSON, L.A., BORST, M. & GOLDMAN, I.D. (1983). Analysis of the role of membrane transport and polyglutamation of methotrexate in gut and the Ehrlich tumour *in vivo* as factors in drug sensitivity and selectivity. *Cancer Res.*, **43**, 1087.

GAUKROGER, J.M., WILSON, L., STEWART, M. & 4 others (1983). Paradoxical response of malignant melanoma to methotrexate *in vivo* and *in vitro*. *Br. J. Cancer*, **47**, 671.

GOLDMAN, I.D. (1975). The membrane transport of methotrexate (NSC-740) and other folate compounds: relevance to rescue protocols. *Cancer Chemother. Rep.*, **6**, 63.

GOLDMAN, I.D., LICHTENSTEIN, N.S. & OLIVERIO, V.T. (1968). Carrier mediated transport of the folic acid analogue, methotrexate in the L1210 leukaemia cell. *J. Biol. Chem.*, **243**, 5007.

JACOBS, S., ADAMSON, R.H., CHABNER, B.A., DERR, C.I. & JOHNS, D.G. (1975). Stoichiometric inhibition of mammalian dihydrofolate reductase by the γ-glutamyl metabolite of methotrexate, 4-amino-4-deoxy-N$^{10}$-methylpteroyl-glutamyl-γ-glutamate. *Biochem. Biophys. Res. Commun.*, **63**, 692.

JACOBS, S.A., STOLLER, R.G., CHABNER, B.A. & JOHNS, D.G. (1976). 7-Hydroxymethotrexate as a urinary metabolite in human subjects and rhesus monkeys receiving high-dose methotrexate. *J. Clin. Invest.*, **57**, 534.

LANKELMA, J., VAN DER KLEIJN E. & RAMAEKERS, F. (1980). The role of 7-hydroxymethotrexate during methotrexate anticancer therapy. *Cancer Lett.*, **9**, 133.

MATHERLY, L.H., FRY, D.W. & GOLDMAN, I.D. (1983). Role of methotrexate polyglutamation and cellular energy metabolism in inhibition of methotrexate binding to dihydrofolate reductase by 5-formyl-tetrahydrofolate in Ehrlich ascites tumour cells *in vitro*. *Cancer Res.*, **43**, 2694.

SCHNEIDER, W., SCHWENK, H. & BURNKAMM, G. (1977). Characterisation of EBV genome negative null and T cell lines derived from children with acute lymphoblastic leukaemia and leukaemic transformed non-Hodgkins lymphoma. *Int. J. Cancer*, **19**, 621.

WHITE, J.C. & GOLDMAN, I.D. (1976). Mechanism of action of methotrexate IV. Free intracellular methotrexate required to supress dihydrofolate reduction to tetrahydrofolate by Ehrlich ascites tumour cells *in vitro*. *Mol. Pharmacol.*, **12**, 711.